# Sustainable Sanitation Management Tool for Decision Making in Isolated Areas in Brazil

**DOI:** 10.3390/ijerph16071118

**Published:** 2019-03-28

**Authors:** Fernando J. C. Magalhães Filho, Adriane A. F. S. L. de Queiroz, Beatriz S. Machado, Paula L. Paulo

**Affiliations:** 1 Department of Sanitary and Environmental Engineering., Dom Bosco Catholic University, Mato Grosso do Sul, Campo Grande 79117-900, Brazil; beatrizsantos.esa@gmail.com; 2 Faculty of Engineering, Architecture, Urbanism and Geography, Federal University of Mato Grosso do Sul, Campo Grande, Mato Grosso do Sul 79070-900, Brazil; adriane.queiroz@gmail.com (A.A.F.S.L.d.Q.); paula.paulo@ufms.br (P.L.P.)

**Keywords:** rural area, software, framework, participatory sanitation programming, oriented-resource sanitation

## Abstract

There is a worldwide range of technical sanitation guidelines focusing on small or traditional and isolated communities for ecological alternatives at the household level. However, a computational tool (software) that has a database and connects these guidelines in a single reference for resource-oriented sanitation concept decision making is still lacking. In this regard, an easy-to-use tool was developed using a participatory approach for the decision-making process from a choice of technical solutions to a type of system management. The results obtained from a pilot study indicate that the proposed tool in this paper will help with the decision-making process to aid in not only choosing sustainable sanitation solutions, but also sustainable operation and maintenance options for the systems. When presenting and discussing the tool with research groups and technicians, the potential for participatory application was noticed. The proposed tool can be used in the elaboration of municipal sanitation plans, assisting local technicians and environmental licensing agencies, designers and engineering students, among others. The software can be applied with other management tools, such as 5W2H and Canvas business model.

## 1. Introduction

Poor water, sanitation, and hygiene (WaSH) cause an estimated 577 thousand deaths annually [1]. Only one-third of countries have human resource strategies for WaSH [2]. WaSH includes a lack of soft skills among program managers such as partnerships and supervision [3], which are increasingly important given a shift in WaSH interventions towards participatory behavior-change approaches that necessitate these skills [4]. Key aspects lacking in WaSH programs that are participatory involve capacity building or target behavior-change. Improving this as a priority, such as meeting the Sustainable Development Goals (SDGs) will require efforts to shift the means of implementation toward capacity building, local participation and behaviors [5].

In general, implemented sanitation projects that do not work tend to fail because decisions are taken without the concerned population’s participation or without considering local conditions or aspirations [6,7,8,9,10,11]. Implementation of wastewater treatment systems cannot be seen as the sole objective of a project. Sanitation provision in the form of infrastructure subsidies or direct investment may not be effective at reducing diarrheal disease risk if good hygiene behaviors are not promoted, [12,13] and stakeholders should not be limited by the provision of local infrastructure solely from authorities. This struggle is clearly one of the main challenges in providing sustainable sanitation and managing activities [14,15,16]. Engaging stakeholders in the proposal selection is essential for the success of the objective [17,18], and community involvement is often the only solution to solving solid or liquid waste problems in low-income areas. In this sense, a community’s awareness and willingness to participate are important aspects to consider when planning and implementing sanitation alternatives. It is fundamental that water and sewage service management allow community involvement, motivating and gaining trust in decision-making. In isolated communities, this participatory management is essential, and information pertaining to the community as well as their wishes should be considered in the decision-making process.

Regarding planning and decision-making processes aimed at sustainable sanitation solutions, guides, manuals, and tools developed by multidisciplinary groups exist. IWA Sanitation 21 [19], for example, aims to support municipal and local authorities in preparing municipal sanitation plans in a rational and realistic manner. The EAWAG-SANDEC Compendium of Sanitation Systems [20] was developed in the context of sustainable decentralized environmental sanitation, being possible to use in a participatory planning process. It groups a wide range of technologies into a single volume, helping users to understand and work with various options to define which sanitation system they should implement. There is also the SSWM toolbox (Sustainable Sanitation and Water Management), the most comprehensive collection of technologies for sustainable sanitation and water management [21]. Its main advantage is its holistic approach; it assists in identifying local problems, supports the planning process and presents numerous solutions that are likely to improve a local situation. Although there is extensive literature on the subject (mainly in English and Spanish), there are still few studies and documents in Portuguese.

Existing guidance in Brazil on sanitation technologies provides general notions about construction, operations, and maintenance. However, they are not suitable for participatory management or for focusing on water reuse and nutrient recovery, particularly in traditional, isolated, and rural communities. Some examples include the FUNASA (National Health Foundation) sanitation manual—which deals with water supply, domestic sewage, rainwater drainage and solid waste management [22]—and the guide for operational and maintenance aspects of septic tank sludge using constructed wetlands [23]. Recently, a book with health improvement guidelines focusing on residential sanitation [24] has been developed, which complements technical guidelines for the elaboration of residential health improvement projects manual [25]. Both were developed by FUNASA, which is responsible for traditional and isolated communities in Brazil [26]. Traditional communities are composed of people or groups that are culturally differentiated and recognized as such. They have their own forms of social organization, which occupy and use territories and natural resources as a condition for their cultural, social, religious, ancestral, and economic reproduction, using knowledge, innovations and practices generated and transmitted by tradition [27]. They are generally isolated in rural areas, far from cities and with no access to conventional transport, energy, water and sanitation infrastructure.

Concurrently, there are computational tools that aid with decision making for sanitation technologies, such as: Water and Wastewater Treatment Technologies Appropriate for Reuse Model (WAWTTAR) [28], SANEX^TM^ decision support system [29], Selection Tool for Natural Wastewater Treatment Systems (SETNAWWAT) [30], and Decision Support System (DSS), which selects sanitation technologies and assists in the planning process in emergencies situations [31], elaborated for specific applications. These computational tools tend to have exhaustive numbers of criteria that hinder the user when there is not a large amount of information about the community being studied. They are neither flexible nor have user-friendly interfaces. In addition, they lack management tools for implementation, operation, maintenance and do not create a value proposition to obtain resources from technologies that reuse water and recovery nutrients. To be more convenient, these tools must be capable of inserting and removing appropriate criteria as well as technologies and have the possibility of proposing sanitation arrangements, which is only possible with a database connection.

Aiming to propose a user-friendly tool to assist in selecting technical and management solutions for sanitation services in isolated areas, the objective of this work was to present the development and application of a tool. This takes into consideration the selection of technically acceptable solutions for the decision-making process in a participatory approach, using management tools such as 5W2H and Canvas with communities. In addition, the tool considers two interventions: the first being with sanitation professionals, and the second with householders.

## 2. Materials and Methods 

The study was developed in 2 stages, the sustainable sanitation management tool development (Table 1) and the tool application in a pilot study in Furnas do Dionísio Quilombola community in Brazil. This case study application was established by an agreement between the university (rectory council), the local energy concessionaire (president of ENERGISA) and an association of residents from the Quilombola community (community leader and residents). This process and the legal mechanisms adopted with the community have the consent of the ethics committee of the university connected to the rectory council—this includes meeting minutes and terms of consent for the use of photos and data by the community leader.

The tool was developed in 4 sections: (a) building the concept of a sustainable sanitation management tool; (b) developing a database (PostGreSQL) to analyze basic sanitation situations related to water supply and domestic sewage; (c) selecting technologies and decision criteria; (d) creating a computational program (Java) with a decision tree based on analysis from diagnosis (class form) with system and alternative technologies (decision criteria).

### 2.1. Software Design: The Concept of a Sustainable Sanitation Management Tool

**Figure 1:**
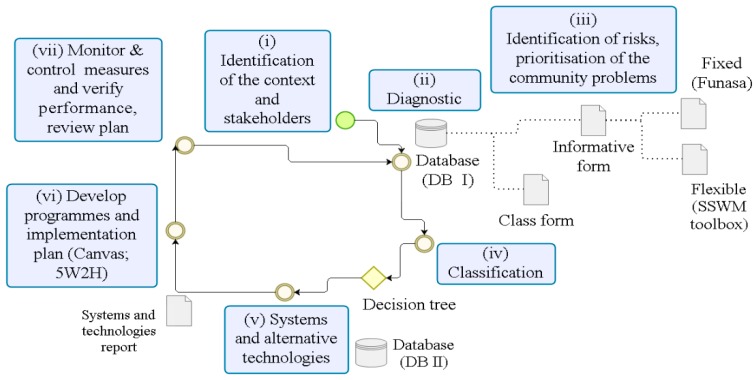
Modules of the sustainable sanitation management tool.

The concept of a sustainable sanitation management tool (Figure 1) was based on the integration of Sanitation 21 [19] and Sanitation Safety Planning [32], in addition to the Term of Reference from FUNASA for sanitation plans [33]. The tool can be applied in different contexts, as a residence, condominium, neighborhood or even a city; and, it includes stakeholders and raises information to diagnose and identify risks to choose appropriate technologies.

The first step is to identify stakeholders (step i) who will carry out the diagnostic (step ii). The diagnostic process (database I—DB I) follows recommendations from the Sustainable Sanitation and Water Management toolbox—SSWM toolbox [24].

### 2.2. Database Management: Diagnostic Process, Selection Criteria, Systems and Alternative Technologies

The diagnostic process has two forms: informative (step iii) and class (step iv). The informative form is divided into two sections: fixed and flexible. The fixed section allows the user to generate graphs and perform statistical analysis with information from several households. The flexible section allows the user to insert different types of questions such as multiple choice, single choice, and descriptive questions in addition to attaching photos and coordinates. The class form (classification) is used by a decision tree to select appropriate technologies by selection criteria.

The sanitation systems and technologies selection criteria (step v) are based on “Compendium” [20], “Microbial Exposure and Health Assessments in Sanitation Technologies and Systems” [34], and “Greywater Management” [35]. These criteria, which include the NBRs (Brazilian Technical Standards) and case studies from FUNASA and PROSAB (National Research Program on Basic Sanitation/Brazil) compiled into an innovative catalog for Brazil—the first criteria for resource-oriented sanitation—is called “CataloSan” [36].

### 2.3. Decision Tree and Participatory Approach

**Figure 2:**
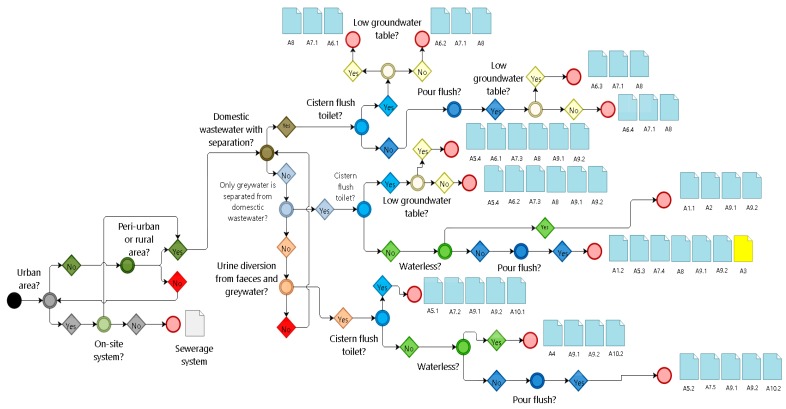
Decision tree based on class form linked to the database (BD II) with sanitation systems.

The decision tree (Figure 2) intersects class form from the diagnostic (DB I—classification) with selection criteria adopted to obtain possible sanitation systems and alternative technologies (Table 2)—first interventions, with sanitation professionals (databases II—DB II). After generating a report with sanitation systems and technologies, a workshop is conducted through a participatory methodology to choose the most appropriate option according to the community—secondary intervention with householders, through meetings to discuss sanitation options that are most feasible. Participatory methodology was used to present results obtained from the software application using community-based approaches [37] for reference, starting by encouraging the participants (of different ages) to reflect on health and hygiene in the rural area using slide presentations, and including people with experience in the process to vote and choose the best option with flipcharts. Therefore, the community would be able to select and implement sanitation systems that are appropriate in their own view using Canvas methodology and 5W2H.

In the technology database (DB II), there is information about technical options (implementation, operation, and maintenance aspects); environmental aspects (groundwater level, rocky soil/area, and digging/excavation difficulty, etc.); cultural aspects (urine use in the soil, squat defecation, and religious aspects regarding water, feces, and urine, among others). Details pertaining to sizing criteria, suitability of region and location, health and acceptance aspects by members, operation and maintenance, advantages, and disadvantages are presented and discussed with local members.

### 2.4. Implementation Plan: Selection of Sanitation Systems by Furnas do Dionísio Quilombolas Community

For the implementation of the chosen sanitation system, Canvas methodology was used as the business model (or Canvas BM) and 5W2H as the management tool (step vi) in order to build an implementation plan.

Canvas BM was used in the context of this study, which is defined as “the rationale of how an organization creates, delivers, and captures value”. Which includes 3 key aspects: (1) How key components are integrated to deliver value to the customer; (2) How those parts are interconnected (stakeholder networks); and (3) How the key components generate value through those interconnections [38,39]. 5W2H (what, when, who, where, why, how, how much) is a very simple and effective quality tool used in cleaner production for describing planned actions in a careful and objective way, thereby ensuring its organized execution providing priority actions through questioning. It consists of equating the problem describing it in the following aspects: what will be done (steps); how it will be done (methods); why it should be done (justification); where each step will be performed (site/place); when the task will be executed (time); who will perform the task (responsibility); and how much each task will cost (cost) [40,41].

It should be noted that a diagnostic database could be used for information, such as indicators, to be stored in order to evaluate communities’ performances and to control whether sanitation plans have been effective (step vii).

### 2.5. Pilot Study in the Furnas do Dionísio Quilombola Community

A pilot study was carried out in the Furnas do Dionísio community. Furnas do Dionísio is a rural black Quilombola community, where 58% are women, and 42% are men, with a mean age of 49.3 years. Located south of the municipality of Jaraguari, approximately 43 km from Campo Grande, capital of Mato Grosso do Sul (a mid-western state in Brazil). They have occupied a permanent piece of land since the end of century XIX, with around 100 families on small plots of land and farms, spanning across 1,018.2796 ha. The local economy is based on agriculture, livestock and tourism. Septic tanks are the only technology used to treat domestic sewage. However, they are not properly constructed and installed, only absorbent and rudimentary. This ends up contaminating the water table, which is a source of water for human consumption, especially in periods with a lack of water in the centralized water supply system. In addition, there is still open-air sewage, from washing clothes and kitchen sinks.

To demonstrate use and application of the developed tool using data collection (diagnostic) through the selection of technological arrangements for domestic sewage, a sanitation system for a community center was designed, where residents get together for social and business activities and receive tourists. To identify stakeholders, a diagnostic was made through informative class forms, and the software was used for the first intervention and participatory approach through the second intervention, and to select alternative technologies.

Considering stakeholder participation relevance in choosing a sanitation arrangement, Canvas BM was used. Then a selection of technical solutions to better understand the community’s value proposition view was implemented followed by 5W2H management tool to build an implementation plan. To understand the local business model, the following steps were adopted to finalize the project: (1) meetings with the community to present resource-oriented sanitation solutions; (2) software use by technical team to select the first set of technologies; (3) workshop with the community using participatory methodology to choose arrangements and technologies; (4) consultation of technology information in DB II; and (5) use of 5W2H management tool for implementation.

The pilot study required a day to collect data with the community leader and one more day (afternoon period) to carry out the participatory methodology for the community to choose and elaborate the implementation (5W2H) and business model (Canvas).

## 3. Results and Discussion

### 3.1. Application and Validation of Sustainable Sanitation Management Tool

**Figure 3:**
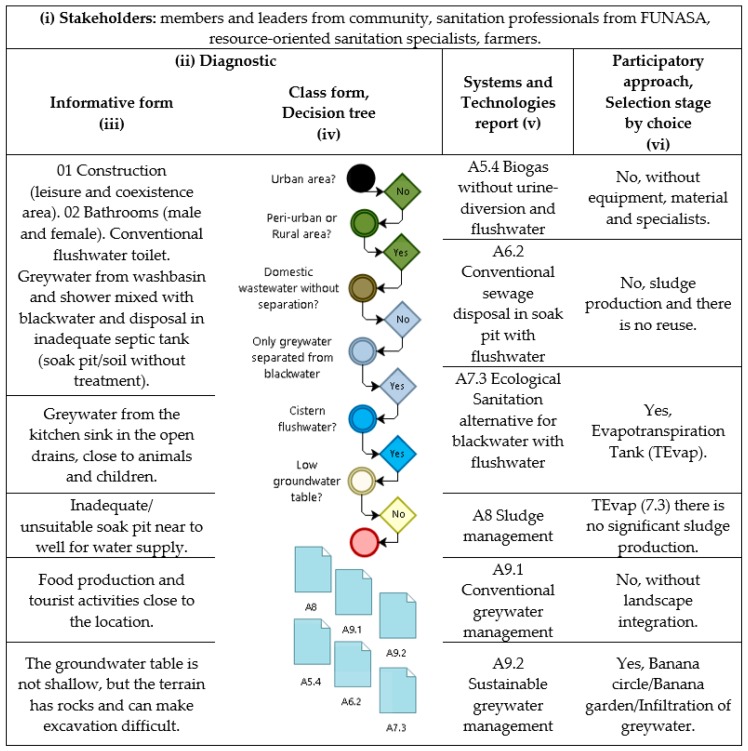
Stakeholder’s identification, diagnostic, decision tree, sanitation systems and participatory approach.

Figure 3 shows a schematic frame with process steps that were followed using the developed tool for the pilot study in Furnas do Dionísio Quilombola community. The stakeholders were identified (i), from the members of the community to the FUNASA team that works with the community. The diagnosis (ii) has an informative (iii) and class (iv) form that describes the pilot study site. From the class form, the decision tree selected alternative technologies and sanitation systems (v), which were approved by the technicians. Finally, the technologies were presented and using the participatory methodology, the members voted and chose the appropriate option, developing an implementation plan with Canvas BM and 5W2H (vi), being an evapotranspiration tank for blackwater and banana circle for greywater.

In a survey study in rural Quilombola communities in Brazil [42], it was observed that there is no participatory management model in decision-making processes for choosing alternative technologies for water and wastewater treatment. Since there is currently only one sanitation system model, based on groundwater collection, which lacks operation and maintenance with no adequate treatment and the only alternative is a dry or absorbent pit, with no possibility of reuse or nutrient recovery, it is impossible to find a sustainable sanitation system. This indicates that an individual household evaluation of water supply and sewage conditions is important. There are construction and plumbing differences between the houses, which allows several technological options within the same community, such as opting for domestic sewage treatment or separating greywater and blackwater. In this sense, a pilot study was also efficient in evaluating participatory methodology, which was widely accepted by the community and showed positive results regarding the consensus of the chosen technologies. If the management tool did not have the second intervention, conventional technologies with the production of sludge and without nutrient recovery could be chosen by technicians, since they have standardized and are already more accepted in the region.

In the first intervention after applying the software (Intervention I—Technique), a biodigester system was discarded due to difficult construction and operation (cost and labor, among others). Systems without water (dry) were suggested by sanitation professionals and consultants even though it was not a result of the software, in order to reduce water consumption in the community. However, there was no interest from members (Intervention 2—Community), as they are culturally used to flushing toilets, being the preference of the majority. In addition, they receive visitors and tourists who are not accustomed to dry systems. Thus, among the technological options, the community opted for evapotranspiration of blackwater, by using a TEvap (Evapotranspiration Tank), combined with a banana tree garden for the greywater fraction. The choice was based on the interest to reuse water and take advantage of nutrients, as well as to provide a more harmonious landscape environment, attractive for tourism activities inherent to the location. A lower operation and maintenance demand with easy implantation of materials, equipment and local labor, without generating sludge, odor and vectors, common to other systems and their technologies was also observed. 

### 3.2. Sustainable Sanitation Management Tool Potential with 5W2H and Canvas Business Model

The result of the Canvas BM application shows the community’s value proposition view (Table 3). It was verified that, after clarifying the common value proposition, the community could decide upon other elements regarding customer segments, customer relationships, and channels, ensuring that the choice of this sanitation arrangement was an important factor. After that, the community could also understand how to keep this value by identifying key activities, key resources, and key partners. The last step was to understand its viability by considering revenue (coming from the value proposition deliver) and costs (derived from building and maintaining value). 

The tool allowed the community to perceive not only the value of the chosen solution, but also to have a broad system view—from implementation to maintenance, considering viability implications to choose a solution. In addition, it is explicit how sanitation provisions, in addition to improving health and environmental conditions, help communities in promoting tourism in their region, as it guarantees adequate conditions to receive visitors, improving the local economy, a fact shown by [43].

After the Canvas BM application, the community discussed a plan about how to implement a sanitation system considering 5W2H and defined a plan for each needed action: what to do, why, where, when, who (is responsible for the action), how and how much (it will cost). 

In order to diffuse and share software, there is a need to invest in key resources (technicians and consultants), and resources used for conventional systems (septic tank, soak pit) can be reverted to the options chosen in a participatory way (revenue streams). It is necessary to follow up on activities with health and environmental education workshops and programs, as suggested by [44] and detailed in the business model under customer relationships and channels.

In Brazil, management models for sanitation have been discussed, including immediate concern to solve local sanitation services’ low attention to rural areas [45]. The National Plan for Basic Sanitation (PNSB) and National Plan for Rural Sanitation (in development) show how evident the need to improve these conditions is by seeking alternative technologies and tools to help sanitation management models.

The tool developed in this study, independent from the public or private model, provides a participatory decision-making framework, guaranteeing that the user feels a sense of belonging and empowerment in relation to water and sewage services, which has been evaluated as essential for the inclusion, participation and organization of communities [44,46].

**Figure 4:**
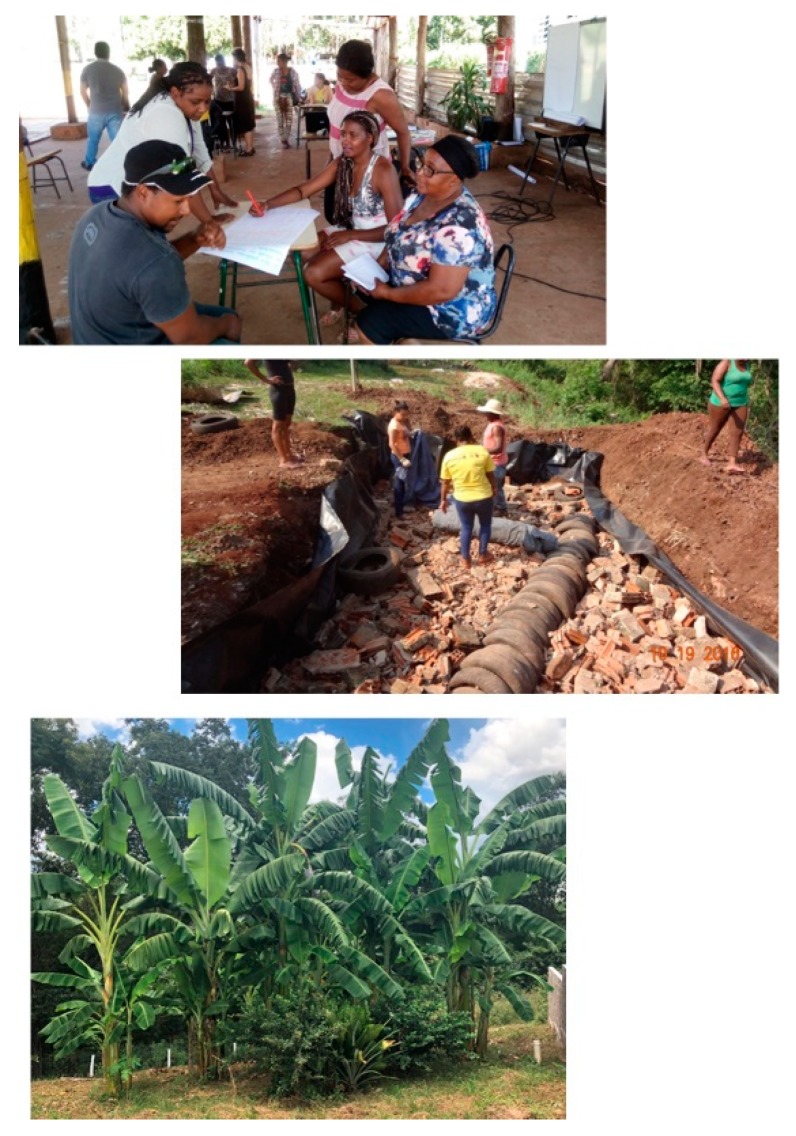
Monitoring & control: decision-making process, implementation of the chosen alternative and 18 months after construction. Consent for publishing these photos was obtained.

To guarantee monitoring and control measure stages and to verify performance, our research group visited every 6 months (Figure 4) to observe the installed system. This confirmed that the community was fulfilling their role with generated effluent for evapotranspiration, that is, with zero discharge. In addition, it was possible to evaluate a better integration of the landscape with the chosen ecological alternatives, allowing a better harmonic environment, providing better tourism conditions in the region and banana production for consumption. The new system also avoids open sewage, which pollutes soil, groundwater, and surface water.

Nevertheless, the management tool is not applied in all contexts; in cases with centralized systems and sewage collection networks, the decision tree does not present alternative technological options in the database, although they can be used. Level and type of community engagement will influence the utility of this tool. Therefore, it will always be important at the moment of the participatory methodology (second intervention), to encourage participants and reflect on health and hygiene situations.

This study presented the management tool applied in a case study; it is suggested that it be applied in other places and situations. However, it would be appropriate for the team that developed the tool to assist in the process. In addition, it would be necessary to have local specialists who know the difficulties of the study area (costs, legislation, and norms), including experiences with ecological technologies and nature-based systems that take advantage of present nutrients.

## 4. Conclusions

This article presents a tool for sustainable sanitation management using database software to assist in the conception of a diagnostic process. In addition, it also guides the solution-making process through a decision tree based on local criteria, appropriate technologies, and sanitation systems with an emphasis on resource-oriented sanitation alternatives. This software can be applied in a home (residence), community building such as a school, shelter for minors, community living space, or an entire neighborhood or community. The diagnosis can identify risks, understand problems, and prioritize actions. The decision tree ensures flexibility by allowing users to insert different technologies and create new sanitation systems, and the information in the database is used during the diagnostic step for the decision process. This tool is database driven and useful for decision making since existing documents that address numerous technologies end up making choices complex, and often designers and technicians make customary decisions.

The proposed methodology allows software to be applied during the elaboration of sanitation plans. An informational database can be used as an indicator to evaluate conditions using a project horizon, analyzing goals, programs, and actions within the sanitation scope. Although this tool was applied to Quilombola communities, it can be used in different contexts—e.g., urban, rural, settlements, slums (favelas), peri-urban areas, among others. This tool provides the need to involve the community in participatory decision-making processes, and it is suggested that the participatory methodology be adapted to local conditions.

A pilot study permitted validation of the applied tool with real results, which enabled the implementation of the chosen sanitation system. Software application demonstrates that communities are able to make decisions about their sanitation systems and choose more sustainable technologies. It became clear that by enabling ecological sanitation alternatives, such as the separation of domestic effluents in this case, communities understand that there are better options than conventional technologies such as septic tanks, enabling the reuse of water and utilization of nutrients present in domestic sewage.

This software can be applied with other management tools, such as 5W2H and Canvas BM, for technology implementation. The proposal of ecological alternatives for resource-focused sanitation presents a more interesting and valuable proposal, and provides gains with users focused on health and environmental education.

## Figures and Tables

**Table 1:** Sustainable sanitation management tool.

The Concept of Tool (Software Design)	Diagnostic Process	Selection Criteria for Sanitation Systems and Alternative Technologies	Participatory Approach and Management Tools
Sanitation 21 [19], Sanitation Safety Planning [32], and Term of Reference from FUNASA for sanitation plans [33].	Sustainable Sanitation and Water Management toolbox—SSWM toolbox [21].	Compendium [20], Microbial Exposure and Health Assessments in Sanitation Technologies and Systems [34], Greywater Management [35], and CataloSan [36]	Community-based approaches [37], Canvas business model [38,39], and 5W2H [40].

**Table 2:** Systems and technologies in database II (DB II).

		Technologies				
Sanitation Systems		User Interface	Collection/ STORAGE	Conveyance	Treatment	Use/Disposal
A1.1	Waterless pit latrine	Dry toilet	Single pit; VIP^3^	-	-	Arborloo
A1.2	Pour flush pit latrine	Pour flush toilet		-	-	
A2	Composting dry toilet	Dry toilet	Double VIP^3^; FA^4^; CA^5^	Human-Powered Emptying and Transport (HPET)	-	Humus and compost
A3	Ecological pit	Pour flush toilet	Twins pit for pour flush		-	
A4	Urine-diverting dry toilet (UDDT)	UDDT	Dehydration Vaults	Motorized Emptying and Transport (MET); HPET	-	Dehydrated Feces
A5.1	Biogas + UDDT + flush toilet (FT)	UDCFT^1^	Biogas reactor (BR)		-	Application of sludge; biogas
A5.2	Biogas + UDDT + pour flush (PF)	UDPFT^2^			-	
A5.3	Biogas + pour flush	Pour flush toilet			-	
A5.4	Biogas + flush toilet	Cistern flush toilet			-	
A6.1	Domestic sewage + wastewater filter strips + FT		Septic tank (ST); ABR^6^	-	-	Infiltration
A6.2	Domestic sewage + soak pit + FT		Septic tank	-	-	
A6.3	Domestic sewage + wastewater filter strips + PF	Pour flush toilet		-	-	
A6.4	Domestic sewage + soak pit + PF			-	-	
A7.1	Ecological alternatives + sewage + FT	Cistern flush toilet		-	CW-HF^7^; CW-FV^8^; sand filter	I^13^; FP^14^; GR^15^; FPP^16^
A7.2	Ecological alternatives + UDDT + FT	UDCFT^1^; Urinal	TEvap; “Fossa” biodigester	-	-	Infiltration
A7.3	Ecological alternatives + blackwater + FT	Cistern flush toilet		-	-	
A7.4	Ecological alternatives + blackwater + PF	Pour flush toilet		-	-	
A7.5	Ecological alternatives + UDDT + PF	UDPFT^2^; Urinal		-	-	
A7.6	Ecological alternatives + sewage + PF	Pour flush toilet	Septic tank	MET; HPET	CW-HF; CW-FV; sand filter	I^13^; FP^14^; GR^15^; FPP^16^
A8	Sludge management	-	-	-	SP^9^; UDB^10^; PDB^11^; C-C^12^; Biogas reactor	Sludge
A9.1	Greywater management	-	Sedimentation tank; ST	-	CW-HF; CW-FV; sand filter	I^13^; FP^14^; GR^15^; FPP^16^; Greywater reuse
A9.2	Greywater sustainable management	-	Banana circle;Mulch filter	-	-	Biomass
A10.1	Yellow water management + FT	UDCFT^1^;	Urine storage tank; MET; HPET		-	Application of urine
A10.2	Yellow water management + PF or waterless	UDDT; UDPFT^2^; Urinal			-	

^1^ UDCFT: urine-diverting cistern flush toilet; ^2^ UDPFT: urine-diverting pour flush toilet; ^3^ VIP: single ventilated improved pit; ^4^ FA: fossa Alterna; ^5^ CA: composting chamber; ^6^ anaerobic baffled reactor; ^7^ CW-HF: constructed wetland—horizontal flow; ^8^ CW-VF: constructed wetland—vertical flow; ^9^ SP: sedimentation ponds; ^10^ UDB: unplanted drying beds; ^11^ PDB: planted drying beds; ^12^ C-C: co-composting; ^13^ I: irrigation; ^14^ FP: fish pond; ^15^ GR: groundwater recharge; ^16^ FPP: floating plant pond.

**Table 3:** Schematic business Canvas business model (BM).

**Key Partners**	**Key Activities**	**Key Resources**	**Valuable Proposals**
FUNASA; community members and leaders; NGOs, technicians from water and sanitation service utilities.	Implement evapotranspiration tank and banana circle; collect greywater; collect fruits, prune and clean plants; produce and sell/consume bananas.	Technicians and sanitation professionals *; construction of sewerage systems; favorable tourism environment.	Promote reuse of water and nutrients from domestic sewage, avoiding contamination and reducing health risks. Improving local landscape and tourism conditions in the community.
**Customer relationships**	**Customer Segments**	**Channels**	**Cost structure and Revenue Streams**
Permanent user guidance on technology use; monitor market of production, sale and consumption of bananas; update data in computational tool.	Furnas community convention center; workers in sugar production.	Regular meetings, radio and cell phone messages; constant use of software; educational and environmental actions with the community.	Resource for household sanitary construction improvements can be used for sustainable sanitation systems; use of local labor at a lower cost.

* 6 Technicians from the National Health Foundation (FUNASA), 3 sanitation professionals from the Federal University of Mato Grosso do Sul and 1 Senior consultant.
